# Simple, Accurate and User-Friendly Differential Constitutive Model for the Rheology of Entangled Polymer Melts and Solutions from Nonequilibrium Thermodynamics

**DOI:** 10.3390/ma13122867

**Published:** 2020-06-26

**Authors:** Pavlos S. Stephanou, Ioanna Ch. Tsimouri, Vlasis G. Mavrantzas

**Affiliations:** 1 Department of Chemical Engineering, Cyprus University of Technology, 30 Archbishop Kyprianou Str., Limassol 3036, Cyprus; 2 Department of Materials, ETH Zürich, CH-8093 Zürich, Switzerland; ioanna.tsimouri@mat.ethz.ch; 3 Department of Chemical Engineering, University of Patras & FORTH-ICE/HT, GR-26504 Patras, Greece; vlasis@chemeng.upatras.gr; 4 Particle Technology Laboratory, Department of Mechanical and Process Engineering, ETH Zürich, Sonneggstrasse 3, CH-8092 Zürich, Switzerland

**Keywords:** entangled polymer melts, concentrated polymer solutions, nonequilibrium thermodynamics, polymer tumbling, transient shear viscosity undershoot

## Abstract

In a recent reformulation of the Marrucci-Ianniruberto constitutive equation for the rheology of entangled polymer melts in the context of nonequilibrium thermodynamics, rather large values of the convective constraint release parameter *β_ccr_* had to be used in order for the model not to violate the second law of thermodynamics. In this work, we present an appropriate modification of the model, which avoids the splitting of the evolution equation for the conformation tensor into an orientation and a stretching part. Then, thermodynamic admissibility simply dictates that *β_ccr_* ≥ 0, thus allowing for more realistic values of *β_ccr_* to be chosen. Moreover, and in view of recent experimental evidence for a transient stress undershoot (following the overshoot) at high shear rates, whose origin may be traced back to molecular tumbling, we have incorporated additional terms into the model accounting, at least in an approximate way, for non-affine deformation through a slip parameter *ξ*. Use of the new model to describe available experimental data for the transient and steady-state shear and elongational rheology of entangled polystyrene melts and concentrated solutions shows close agreement. Overall, the modified model proposed here combines simplicity with accuracy, which renders it an excellent choice for managing complex viscoelastic fluid flows in large-scale numerical calculations.

## 1. Introduction

Using accurate constitutive models to describe the complex rheological response of high molecular weight (MW) polymers (melts or solutions) to an applied flow field allows for the more economic, rational design of new polymer-based products as well as for the improved description of processing operations as these models are key ingredients in large-scale numerical calculations of viscoelastic fluid flows. A successful model should be able to accurately predict changes in the rheology of the material during processing as a function of chemical structure and composition, molecular architecture, and morphology (possible co-existence of crystalline and amorphous domains).

Since its introduction and after several modifications and improvements, the tube model [1,2] has offered a remarkable conceptual framework for formulating theories that can explain but also quantitatively describe the behavior of entangled, high-MW polymer melts and concentrated polymer solutions, both under equilibrium and nonequilibrium (flow) conditions. It relies on the idea that due to chain uncrossability and the development of entanglements with other chains, the lateral motion of a reference chain is effectively constrained within a curvilinear tube-like region (a mean-field tube) around the primitive path of the chain with a diameter whose magnitude reflects the strength of these topological interactions. In the simplest case of a linear chain that is restricted within such a tube-like region, relaxation is achieved through backward and forward diffusion (i.e., through reptation) along the axis of the confining tube [1,2]. Today, chain reptation within the mean-field tube is not simply a mathematical invention; it has been directly observed in molecular simulations [3]. However, it is also well-known that for the tube model to be able to quantitatively describe the equilibrium dynamics of actual polymeric systems, it has to account for additional mechanisms, such as contour length fluctuations (CLFs) due to the “breathing” motion of chain ends, and constraint release (CR) due to the destruction of topological constraints as a result of the simultaneous (collective) relaxation of the other chains surrounding the reference chain.

A mechanism that the original theory failed to consider under strong flows is that of convective constraint release (CCR). In this case, when the shear rate exceeds 1/*τ_d_* where *τ_d_* is the inverse chain reptation or disentanglement time, the simultaneous motion of the surrounding chains comprising the mean-field tube leads to the release of entanglements, thus inducing a faster relaxation [4,5]. Self-consistently, this means that the number of entanglements should be a decreasing function of the strength of the applied flow, a notion that was originally missed by polymer physicists and rheologists. This has been directly demonstrated in the nonequilibrium molecular dynamics (NEMD) simulations of Baig et al. [6] with an entangled polyethylene melt under shear flow, as well as in several other NEMD simulation studies afterwards [7,8,9,10]. The direct observation of flow-induced chain disentanglement was incorporated a few years later into a novel and very successful constitutive rheological model by Ianniruberto and Marrucci accounting explicitly for the CCR mechanism [11]. Chain stretching was also considered [12] by proposing two separate evolution equations: one for the orientation tensor **S** describing the average orientation of tube segments, and one for the scalar variable *λ* accounting for the average chain stretch. However, Wapperom et al. [13] argued that such a model leads to anomalous behavior when employed in numerical simulations of complex flows. This motivated Ianniruberto and Marrucci [14] to propose a suitable modification that unified the two different equations into a single one through the use of a single conformation tensor.

Another important mechanism that was originally neglected but was again pointed out by NEMD simulations to be of relevance is rotation or tumbling even of entangled chains under shear [6,7,8,15]. Shear-induced tumbling of entangled polymer chains was incorporated into a constitutive model by Costanzo et al. [16] by introducing a “tumbling” function. In fact, the authors showed that this tumbling behavior is responsible for the appearance of a transient stress undershoot (following the overshoot) at high shear rates in the case of polymer solutions but not in the case of polymer melts. A similar conclusion was reached by Stephanou et al. [17,18] who revisited the kinetic theory of Curtiss-Bird [19,20,21] and solved the corresponding model equations [22] in the presence of rotational diffusion. They showed that the tumbling-snake model for polymeric systems, as they coined the kinetic theory of Curtiss-Bird, predicts pronounced undershoots in the time-dependent shear viscosity (arising from the rotational Brownian diffusion term in the equation of motion for polymer segments) but not in the transient normal stress coefficients. Obviously, such undershoots should be absent in the transient extensional viscosities, since elongational flows are by default free of any rotational contributions to the velocity gradient tensor, and this was indeed verified to be the case [16,23,24]. Additional results from NEMD simulations demonstrated that the overshoot and undershoot in the shear viscosity upon flow startup are correlated to tube orientation and chain tumbling, respectively [9,10].

In a recent contribution [25], we formally re-derived the Ianniruberto-Marrucci [14] and Rolie-Poly [26] models in the context of the generalized bracket [27] and GENERIC [28,29,30] formalisms of nonequilibrium thermodynamics (NET), and appropriately extended them to account, in a self-consistent manner, for a second normal stress difference. However, by applying the 2nd law of thermodynamics and requiring the evolution equation to preserve the positive -definite nature of the conformation tensor between successive entanglement points along a chain for all times and all flow fields was found to constrain the so-called CCR parameter *β*_ccr_ to rather large values (larger than one), which seems to be too restrictive. In the present article, we provide a reformulation of that model that allows the use of more realistic values of the CCR parameter. We also introduce in the model terms that account for non-affine motion of entanglement strands (allowing for chain tumbling) through a slip parameter *ξ*. The use of nonequilibrium thermodynamics guarantees that this extra mechanism is self-consistently incorporated in the constitutive model, which also ensures the internal consistency of the final set of transport equations.

The structure of the rest of the paper is as follows: the nonequilibrium thermodynamics formulation of the new (refined) model in the context of the generalized bracket formalism [27] is presented in the next section; the proof of its thermodynamic admissibility is detailed in Appendix A. We note here that at the level of description of interest in this work, the generalized bracket, and the GENERIC [28,29,30] formalisms are equivalent. In Section 3, we derive asymptotic solutions of the model in the cases of steady-state and transient shear and elongational flows, in the limit of weak flows. We also discuss how closely the new model can describe experimental rheological data already reported in the literature over the years both for entangled polymer melts and concentrated polymer solutions. We conclude with Section 4 presenting a summary of the most important results of our study and a brief discussion of future plans.

## 2. Methods

### 2.1. Nonequilibrium Thermodynamics Refinement of the Stephanou et al. Model

Following Stephanou et al. [25], in the modified version of our model, we also make use of only one structural parameter, the entanglement strand conformation tensor **c**, which is made dimensionless through $\tilde{c}=K\;c/k_{B}T$ where *K* denotes the spring constant of the Hookean dumbbells representing entanglement strands, *k_B_* the Boltzmann constant, and *T* the absolute temperature. The conformation tensor $\tilde{c}$ refers to one entanglement strand and at equilibrium (zero flow field applied) coincides by definition with the unit tensor **I**. Contrary to our previous model, however, we consider an extra contribution to the dissipative bracket, in addition to relaxation phenomena at the level of the structural variable, namely strand non-affine motion, introduced through a fourth-rank tensor **L**. Then, using the well-known procedure of the generalized bracket formalism [27], the resulting evolution equation for **c** reads:

(1) $${\dot{c}}_{\alpha \beta ,[1]}=-\Lambda_{\alpha \beta \gamma \varepsilon }\frac{\delta A}{\delta c_{\gamma \varepsilon }}+L_{\alpha \beta \gamma \varepsilon }\nabla_{\gamma }\frac{\delta H_{m}}{\delta M_{\varepsilon }}$$

where, on the left hand-side, we have defined the upper-convected Maxwell time derivative:

(2) $${\dot{c}}_{\alpha \beta ,[1]}\equiv \frac{\partial c_{\alpha \beta }}{\partial t}+u_{\gamma }\nabla_{\gamma }c_{\alpha \beta }-c_{\alpha \gamma }\nabla_{\gamma }u_{\beta }-\nabla_{\gamma }u_{\alpha }c_{\gamma \beta }$$

In Equation (1), *A* denotes the Helmholtz free energy of the system, *H_m_* the mechanical part of the system Hamiltonian, Λ the relaxation fourth-rank matrix, and **L** the fourth-rank matrix associated with the non-affine motion mechanism. The corresponding expression for the stress tensor is [27].

(3) $$\sigma_{\alpha \beta }=2c_{\gamma \beta }\frac{\delta H_{m}}{\delta c_{\alpha \gamma }}+L_{\alpha \beta \gamma \varepsilon }\frac{\delta H_{m}}{\delta c_{\gamma \varepsilon }}$$

In Equations (1)–(3), we have assumed Einstein’s implicit summation convention for any repeated Greek indices (and this will be followed throughout this work). We will also restrict our analysis to incompressible and isothermal fluids.

The mechanical part of the Hamiltonian is simply equal to the kinetic energy plus the Helmholtz free energy:

(4a) $$H_{m}=\int \frac{M^{2}}{2\rho }dV+A\left(\tilde{c}\right)$$

The latter is given as [25,31,32]

(4b) $$A(\tilde{c})=\frac{G_{e}}{2}\int \left[\Phi \left(\mathrm{tr}(\tilde{c}-I)\right)-\ln\det\tilde{c}\right]dV$$

and includes two terms: the dimensionless potential $\Phi \left(\mathrm{tr}(\tilde{c}-I)\right)$ accounting for chain stretching, and an entropic contribution involving the logarithm of the determinant of the conformation tensor. In Equation (4b), *G_e_* = *n_e_k_B_T* is the entanglement modulus with *n_e_* being the entanglement density (assumed to be constant, independent of the flow). The partial derivative of the potential $\Phi \left(\mathrm{tr}(\tilde{c}-I)\right)$ with respect to the trace of $\tilde{c}$ defines the (dimensionless) effective spring constant *h* [27,30,33]:

(5) $$h\left(\mathrm{tr}\left(\tilde{c}-I\right)\right)=2\frac{\delta \tilde{A}\left(\tilde{c}\right)}{\delta \mathrm{tr}\left(\tilde{c}-I\right)}=\frac{\partial \Phi \left(\mathrm{tr}(\tilde{c}-I)\right)}{\partial \mathrm{tr}\left(\tilde{c}-I\right)}$$

where with $\tilde{A}=A/G_{e}$ we have denoted the dimensionless free energy. The corresponding Volterra derivative of the dimensionless free energy with respect to $\tilde{c}$ is

(6) $$\frac{\delta \tilde{A}\left(\tilde{c}\right)}{\delta \tilde{c}}=\frac{1}{2}\left[h\left(\mathrm{tr}\left(\tilde{c}-I\right)\right)I-{\tilde{c}}^{-1}\right]$$

In Ref. [25], we had employed the FENE-P (Cohen) approximation [34] rather than the FENE-P (Warner) one, as it is a better approximation to the inverse Langevin function [33,34]. However, Varchanis et al. [35] found that the FENE-P (Cohen) force law leads to two different solutions, both stable and both preserving the positive definite nature of the conformation tensor, with one, however, being aphysical. Motivated by this finding, in the present study we chose to work with the FENE-P (Warner) approximation for which [33]:

(7a) $$\Phi \left(\mathrm{tr}\left(\tilde{c}-I\right)\right)=-\left(b_{e}-3\right)\ln\left(1-\frac{\mathrm{tr}\left(\tilde{c}-I\right)}{b_{e}}\right)$$

(7b) $$h\left(\mathrm{tr}(\tilde{c}-I)\right)=\frac{b_{e}-3}{b_{e}-\mathrm{tr}\tilde{c}}$$

Note that Equation (7a) differs slightly from Equation (23a) in Stephanou et al. [33]: although both equations lead to the same expression for the force law, Equation (7a) vanishes at equilibrium as it should, whereas Equation (23a) in Stephanou et al. [33] does not. In the above expressions, $b_{e}\equiv 3L_{e}^{2}/\langle a_{e}^{2}\rangle$ is the extensibility parameter with *L_e_* denoting the maximum length of the entanglement strand. The length of an entanglement strand is equal to *L*/(*Z* + 1), where *L* is the primitive path contour length and *Z* the number of entanglements per chain. For given polymer chemistry, the extensibility parameter should not be considered as an adjustable (free) parameter but rather as a known constant, equal to $b_{e}\equiv \left[3{\left(0.82\right)}^{2}/C_{\infty }\right]\left(M_{e}/M_{0}\right)$ where *C*_∞_ is the polymer characteristic ratio at infinite chain length, *M_e_* the entanglement molecular weight, and *M*_0_ the average molar mass per backbone bond, which is equal to half the monomer molecular weight (note the correction in Appendix A of Ref. [25]). For example, for polystyrene (PS): *M*_0_ = 52 g/mol and *C*_∞_ = 9.6 (Ref. [36], Table 3.3), hence *b_e_* = *M_e_*/298 g/mol. Consequently, for PS melts for which *M_e_* ≈ 13,300 g/mol (Ref. [36], Table 3.3), we find *b_e_* = 54, whereas for entangled polymer solutions the value of *b_e_* will depend on the polymer concentration of the solution (see Section 3.2.2).

In order to be able to predict a transient stress undershoot (following the overshoot) at high shear rates, the new model accounts for non-affine deformation effects in Equation (1) through the tensor ***L***, for which we consider the following form [27]:

(8) $$L_{\alpha \beta \gamma \varepsilon }=-\frac{\xi }{2}\left({\tilde{c}}_{\alpha \gamma }\delta_{\beta \varepsilon }+{\tilde{c}}_{\alpha \varepsilon }\delta_{\beta \gamma }+{\tilde{c}}_{\beta \gamma }\delta_{\alpha \varepsilon }+{\tilde{c}}_{\beta \varepsilon }\delta_{\alpha \gamma }\right)$$

where *ξ* denotes the non-affine or slip parameter. The same form has been used by Beris et al. [37] to capture the thixotropic behavior of concentrated star polymer suspensions.

Finally, the stress tensor is obtained by substituting Equations (6) and (8) into Equation (3), and reads:

(9) $$\sigma_{\alpha \beta }=\left(1-\xi \right)G_{e}\left[h\left(\mathrm{tr}(\tilde{c}-I)\right){\tilde{c}}_{\alpha \beta }-\delta_{\alpha \beta }\right]$$

Defining the relaxation matrix **Λ** is the only remaining task. To this, we consider two relaxation times, the disentanglement or reptation time *τ_d_*, and the Rouse time *τ_R_*, implying that two relaxation matrices should be defined:

(10) $$\begin{array}{l} \Lambda_{\alpha \beta \gamma \varepsilon }^{\tau_{d}}=\frac{f_{\tau_{d}}\left(\mathrm{tr}\tilde{c}\right)}{\tau_{d}G_{e}}\left({\tilde{c}}_{\alpha \gamma }{\tilde{\beta }}_{\beta \varepsilon }+{\tilde{c}}_{\alpha \varepsilon }{\tilde{\beta }}_{\beta \gamma }+{\tilde{c}}_{\beta \gamma }{\tilde{\beta }}_{\alpha \varepsilon }+{\tilde{c}}_{\beta \varepsilon }{\tilde{\beta }}_{\alpha \gamma }\right)\\ \Lambda_{\alpha \beta \gamma \varepsilon }^{\tau_{R}}=\frac{f_{\tau_{R}}\left(\mathrm{tr}\tilde{c}\right)}{2\tau_{R}G_{e}}\left({\tilde{c}}_{\alpha \gamma }{\tilde{\beta }}_{\beta \varepsilon }+{\tilde{c}}_{\alpha \varepsilon }{\tilde{\beta }}_{\beta \gamma }+{\tilde{c}}_{\beta \gamma }{\tilde{\beta }}_{\alpha \varepsilon }+{\tilde{c}}_{\beta \varepsilon }{\tilde{\beta }}_{\alpha \gamma }\right) \end{array}$$

In Equation (10), $\tilde{\beta }$ is the (dimensionless) mobility tensor (see Ref. [25]) and $f_{\tau_{d}}$ and $f_{\tau_{R}}$ scalar functions of the trace of $\tilde{c}$ defined as:

(11) $$f_{\tau_{R}}\left(\mathrm{tr}\tilde{c}\right)=1-f_{\tau_{d}}\left(\mathrm{tr}\tilde{c}\right)=\frac{\beta_{ccr}\left[h\left(\mathrm{tr}(\tilde{c}-I)\right)\mathrm{tr}\tilde{c}-3\right]}{3+\beta_{ccr}\left[h\left(\mathrm{tr}(\tilde{c}-I)\right)\mathrm{tr}\tilde{c}-3\right]}$$

The (total) relaxation matrix Λ needed in Equation (1) is then $\Lambda =\Lambda^{\tau_{d}}+\Lambda^{\tau_{R}}$. It is of interest to discuss how these expressions for the relaxation matrices differ from the expression used in the previous work, Equation (10a) in Ref. [25]. In the previous version of our model, the characteristic relaxation time associated with changes in the stretching of the entanglement segment (i.e., of the trace of the conformation tensor) was the Rouse time, which necessitated that $\Lambda^{\tau_{d}}$ be selected such that $\Lambda_{\alpha \alpha \gamma \varepsilon }^{\tau_{d}}=0$. To avoid imposing strict restrictions on the CCR parameter in the present work (to guarantee the thermodynamic admissibility of the model, see below), the characteristic relaxation time associated with stretching is taken to be the CCR relaxation time, Equation (12c).

Upon inserting Equations (4a), (6), (8), and (10) in Equation (1), we obtain the following evolution equation for the dimensionless conformation tensor:

(12a) $${\dot{\tilde{c}}}_{\alpha \beta ,[JS]}=-\frac{1}{\tau_{ccr}\left(\mathrm{tr}\tilde{c}\right)}\left\{h\left(\mathrm{tr}(\tilde{c}-I)\right){\tilde{c}}_{\alpha \gamma }{\tilde{\beta }}_{\beta \gamma }-{\tilde{\beta }}_{\alpha \beta }\right\}$$

where, on the left hand-side, we have defined the Johnson-Segalman time-derivative:

(12b) $${\tilde{\dot{c}}}_{\alpha \beta ,[JS]}\equiv {\tilde{\dot{c}}}_{\alpha \beta ,[1]}+\frac{\xi }{2}\left({\dot{\gamma }}_{\alpha \gamma }{\tilde{c}}_{\gamma \beta }+{\tilde{c}}_{\alpha \gamma }{\dot{\gamma }}_{\gamma \beta }\right)$$

while the CCR relaxation time is given as:

(12c) $$\frac{1}{\tau_{ccr}\left(\mathrm{tr}\tilde{c}\right)}=\frac{2}{\tau_{d}}+\left(\frac{1}{\tau_{R}}-\frac{2}{\tau_{d}}\right)\frac{\beta_{ccr}\left[h\left(\mathrm{tr}(\tilde{c}-I)\right)\mathrm{tr}\tilde{c}-3\right]}{3+\beta_{ccr}\left[h\left(\mathrm{tr}(\tilde{c}-I)\right)\mathrm{tr}\tilde{c}-3\right]}$$

with *β_ccr_* being the effective CCR parameter. Note that in order for the model to account for both reptation and constraint release (at least in a crude way), we have invoked the *double reptation* approximation and considered the equilibrium CCR relaxation time to be half the corresponding reptation time when only reptation is considered, i.e., ½*τ_d_* [14]. When a non-zero value of the CCR parameter is considered, the CCR relaxation time *τ_ccr_* will exhibit a flow-induced reduction from its equilibrium value (i.e., ½*τ_d_*) towards its corresponding value at large flow rates (i.e., the Rouse time). Finally, for the mobility tensor, we invoke Giesekus’ postulate that $\tilde{\beta }=I+\alpha \;\tilde{\sigma }$ [25,33], where α is the anisotropic mobility parameter and $\tilde{\sigma }=\sigma /G_{e}$ denotes the dimensionless stress tensor. Thus, the evolution equation, Equation (12a), becomes (in the following, for the purpose of simplifying notation, we will drop the arguments in the functions $h\left(\mathrm{tr}(\tilde{c}-I)\right)$ and $\tau_{ccr}\left(\mathrm{tr}\tilde{c}\right)$):

(13) $${\tilde{\dot{c}}}_{\alpha \beta ,\left[\mathrm{JS}\right]}=-\frac{1}{\tau_{ccr}}\left\{\alpha \left(1-\xi \right)h^{2}{\tilde{c}}_{\alpha \gamma }{\tilde{c}}_{\beta \gamma }+\left[1-2\alpha \left(1-\xi \right)\right]h{\tilde{c}}_{\alpha \beta }-\left[1-\alpha \left(1-\xi \right)\right]\delta_{\alpha \beta }\right\}$$

As far as the conditions that should be obeyed in order for the model to be thermodynamically admissible and preserve the positive-definite character of the conformation tensor, these are elaborated in Appendix A and require that:

(14) $$0\leq \alpha \leq {\left(1-\xi \right)}^{-1}\;\mathrm{and}\;\beta_{ccr}\geq 0$$

According to these, the proposed revised model allows for any non-negative value of the CCR parameter to be used without violating the laws of thermodynamics, which should be contrasted with the earlier version [25], wherein *β_ccr_* had to be strictly larger than unity. Finally, note that in deriving the above constitutive model, we have assumed that the contribution of the solvent to the total stress is too small compared to the contribution of polymer (although it can be easily incorporated if needed).

### 2.2. Asymptotic Solutions

We proceed next to analyze the asymptotic behavior of the new model in the limit of weak flows for the following three cases: inception of simple shear flow (SSF) described by the kinematics $u=\left({\dot{\gamma }}_{0}y,0,0\right)$, inception of uniaxial elongation flow (UEF) described by the kinematics $u=\left({\dot{\varepsilon }}_{0}x,-\frac{1}{2}{\dot{\varepsilon }}_{0}y,-\frac{1}{2}{\dot{\varepsilon }}_{0}z\right)$, and small amplitude oscillatory flow (SAOF) described by the kinematics $u=\left(\dot{\gamma }\cos\left(\omega \;t\right)y,0,0\right)$. The material functions to analyze are: a) the shear viscosity *η* ($=\sigma_{xy}/{\dot{\gamma }}_{0}$) and the two normal stress coefficients Ψ_1_ ($=\left(\sigma_{xx}-\sigma_{yy}\right)/{\dot{\gamma }}_{0}^{2}$) and Ψ_2_ ($=\left(\sigma_{yy}-\sigma_{zz}\right)/{\dot{\gamma }}_{0}^{2}$) in the case of shear, b) the extensional viscosity *η*_1E_ ($=\left(\sigma_{xx}-\sigma_{yy}\right)/{\dot{\varepsilon }}_{0}$) in the case of uniaxial elongation, and c) the storage $G^{\prime }\left(\omega \right)$ and loss $G^{\prime \prime }\left(\omega \right)$ moduli in the case of SAOF.

To get asymptotic expressions for the conformation tensor and consequently for the material functions in the limit of small strain rates, we invoke a linearization of the evolution equation for the conformation tensor, which in a second step is solved analytically to also provide the stress tensor components. Then, the following results are obtained for the relevant viscometric or material functions:

Inception of shear:

(15a) $$\eta^{+}\left(t\right)=\eta_{0}\left[1-\exp\left(-2t/\tau_{d}\right)\right]$$

(15b) $$\Psi_{1}^{+}\left(t\right)=\Psi_{1,0}\left[1-\left(1+\frac{2t}{\tau_{d}}\right)\exp\left(-\frac{2t}{\tau_{d}}\right)\right]$$

(15c) $$\begin{array}{r} \Psi_{2}^{+}\left(t\right)=-\frac{1}{2}\Psi_{1,0}\{\left[\xi +\alpha {\left(1-\xi \right)}^{2}\right]\left[1-\left(1+\frac{2t}{\tau_{d}}\right)\exp\left(-\frac{2t}{\tau_{d}}\right)\right]\\ +\alpha {\left(1-\xi \right)}^{2}\exp\left(-\frac{2t}{\tau_{d}}\right)\left[1-\frac{2t}{\tau_{d}}-\exp\left(-\frac{2t}{\tau_{d}}\right)\right]\} \end{array}$$

where *η_0_* = (1-ξ)^2^*G_e_*½*τ_d_* and Ψ_1,0_ = *η_0_τ_d_* are also the zero-rate steady-state values of the shear viscosity and of the first normal stress coefficient:

(16a) $$\underset{{\dot{\gamma }}_{0}\to 0}{\lim}\frac{\eta }{\eta_{0}}=1$$

(16b) $$\underset{{\dot{\gamma }}_{0}\to 0}{\lim}\frac{\Psi_{1}}{\Psi_{1,0}}=1$$

(16c) $$\underset{{\dot{\gamma }}_{0}\to 0}{\lim}\frac{\Psi_{2}}{\Psi_{1,0}}=-\frac{1}{2}\left[\alpha {\left(1-\xi \right)}^{2}+\xi \right]$$

Inception of uniaxial elongation:

(17) $$\eta_{E}^{+}\left(t\right)=3\eta_{0}\left[1-\exp\left(-2t/\tau_{d}\right)\right]$$

and thus, Trouton’s law holds for the steady-state extensional viscosity, i.e.:

(18) $$\underset{{\dot{\varepsilon }}_{0}\to 0}{\lim}\frac{\eta_{1E}}{\eta_{0}}=3$$

Small Amplitude Oscillatory flow:

(19) $$G^{\prime }\left(\omega \right)=\eta_{0}\frac{1}{2}\tau_{d}\frac{\omega^{2}}{1+{\left(\frac{1}{2}\tau_{d}\omega \right)}^{2}};\;G^{\prime \prime }\left(\omega \right)=\frac{\eta_{0}\omega }{1+{\left(\frac{1}{2}\tau_{d}\omega \right)}^{2}}$$

## 3. Results and Discussion

In this section, we will use the FENE-P (Warner) approximation for the function *h* [Equation (7b)]. In Section 3.1, we will also employ the relation *τ_d_* = 3Z*τ_R_* [2], take *b_e_* = 100, and keep the number of entanglements *Z* equal to 20.

### 3.1. Material Functions in Shear

**Figure 1:**
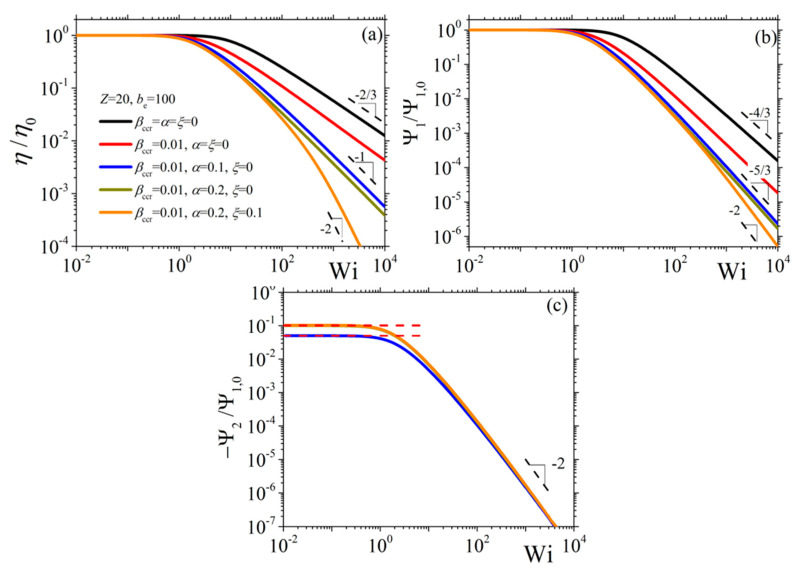
Model predictions for the three material functions (in scaled units) in shear, and dependence on the parameters α, *ξ*, and *β_ccr_*: (**a**) shear viscosity, (**b**) first normal stress coefficient, and (**c**) second normal stress coefficient [the red dashed lines depict the zero-rate value, Equation (16c)].

In Figure 1, we depict the model predictions for the steady-state shear viscosity (Figure 1a) and the first (Figure 1b) and second (Figure 1c) normal stress coefficients (appropriately scaled with the zero-rate viscosity *η*_0_ and first normal stress coefficient Ψ_1,0_), as a function of the applied dimensionless shear rate $\mathrm{Wi}\equiv {\dot{\gamma }}_{0}\tau_{d}$; results for various values of the model parameters are shown. When a vanishing value of the anisotropic parameter *α* is considered, then a vanishing second normal stress coefficient is predicted (the corresponding curves are missing from Figure 1c) As the CCR parameter increases, both *η* and Ψ_1_ exhibit a faster shear thinning behavior, which is the expected behavior given that the CCR parameter introduces a shear-induced decrease of the relaxation time.

However, and irrespective of the value of the CCR parameter assumed, the power-law behavior at large shear rates remains unaltered, with the corresponding exponents equal to −2/3 for the shear viscosity and equal to −4/3 for the first normal stress coefficient. Then, while keeping *β_ccr_* constant, upon increasing the value of α from 0 to 0.1, the power-law exponent at large shear rates changes to −1 and −5/3 for *η* and Ψ_1_, respectively. By further increasing the value of α to 0.2, the shear viscosity is affected only slightly, while the first normal stress coefficient is not affected at all. In the case of the second normal stress coefficient, we note a non-vanishing value, which at small shear rates reaches the zero-rate value dictated by Equation (16c). Thus, as α increases, the zero-rate value increases. However, the large shear rate power-law exponent remains equal to −2 irrespective of the values of the parameters. Finally, for non-zero values of the non-affine parameter *ξ*, we note that until about Wi = 100, the predictions for *η* and Ψ_1_ are the same as when *ξ* = 0, but after that the power-law behavior differs, the corresponding exponent for both *η* and Ψ_1_ becomes now equal to −2. On the other hand, the prediction for Ψ_2_ remains the same.

**Figure 2:**
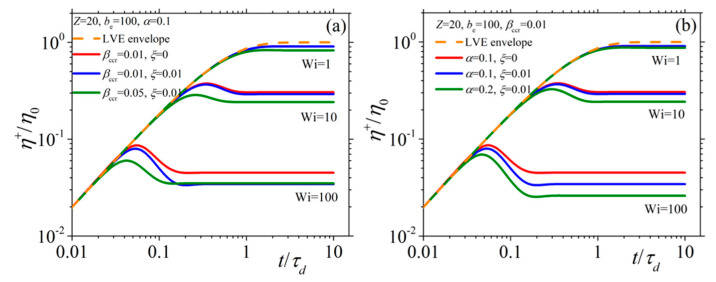
Growth of the shear viscosity upon inception of the shear flow at different dimensionless shear rates for several values of the model parameters (α, *ξ*, and *β_ccr_*) when (**a**) keeping α constant, and (**b**) keeping *β_ccr_* constant. The LVE envelope is depicted as a dashed orange line.

**Figure 3:**
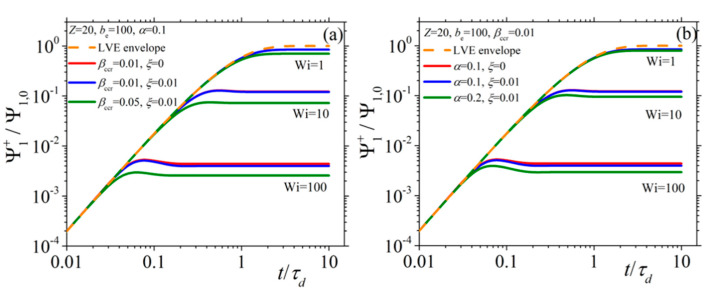
Same as with Figure 2 but for the first normal stress coefficient when (**a**) keeping α constant, and (**b**) keeping *β_ccr_* constant.

**Figure 4:**
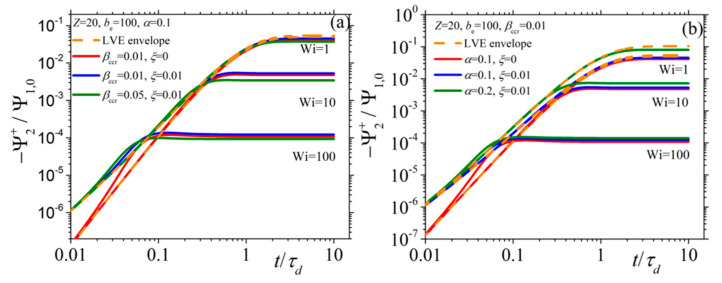
Same as with Figure 2 but for the second normal stress coefficient when (**a**) keeping α constant, and (**b**) keeping *β_ccr_* constant.

In Figure 2 and Figure 3, we present the model predictions for the growth of the shear viscosity and the first normal stress coefficient, respectively, upon inception of the shear flow at three different values of the dimensionless shear rate Wi (= 1, 10, and 100), along with the prediction of the LVE behavior given by Equations (15) (dotted orange line). Irrespective of the shear rate and the values of the parameters assumed, at sufficiently short times, all viscometric curves follow the LVE prediction, which is the expected theoretical prediction. At small shear rates (Wi = 1), both functions are seen to approach their steady-state values monotonically, whereas at larger shear rates (Wi = 10), they go through an overshoot before they reach their steady-state values. Finally, at large shear rates (Wi = 100) and for a non-zero value of the non-affine parameter, the shear viscosity goes through an undershoot right after the overshoot, which is more pronounced in the curves of Figure 2b. Of course, this is only a rather small undershoot; more pronounced undershoots will be reported in the next section, when we will discuss how the model can fit true experimental data (see Figures 7a and 11b). Furthermore, note that both the intensity and the position of this undershoot are controlled by the parameters of the model. Such a behavior is not noticed for the first normal stress coefficient. Overall, the same trend is noticed also for the second normal stress coefficient (Figure 4) whereas, contrary to the other two viscometric functions, the transient curves go over (instead of below) the LVE predictions. Overall, it is a remarkable attribute of the new, single-mode viscoelastic model that it can predict undershoots in the transient shear viscosity.

### 3.2. Comparison with Experimental Data for Entangled Polystyrene Melts and Solutions

#### 3.2.1. Comparison with the Experimental Data Presented in Stephanou et al.

**Figure 5:**
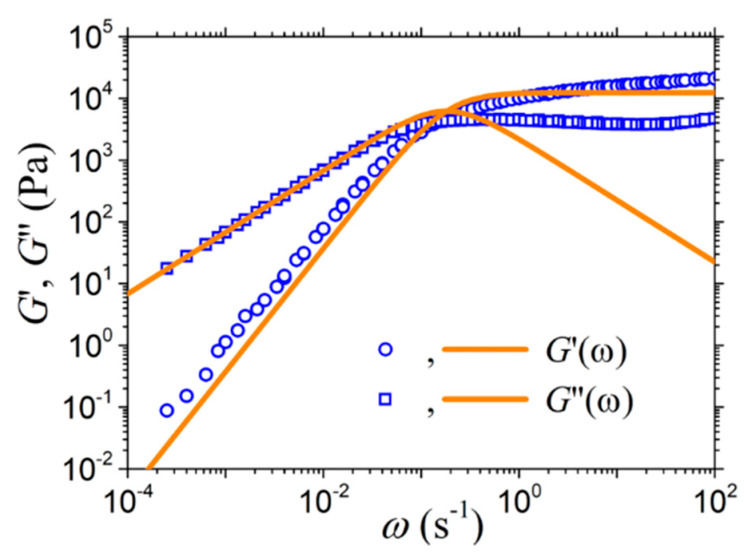
Comparison of the model predictions with the experimental data considered in Ref. [17] for the storage and loss moduli at 30 °C of a nearly monodisperse PS solution with *Μ* = 899 kDa and polymer volume fraction 36.7% (Z = 13.9) characterized by *τ_d_* = 11 s and *η*_0_ = 68 kPa.s.

Figure 5 shows how the new model can fit the experimental data for the spectra of the storage and loss moduli discussed in Ref. [17] in conjunction with the earlier version of this model; the model predictions for both model variants are those presented in Equation (19). To carry out the fitting, we first need to specify two of the model parameters: the reptation time *τ_d_*, and either the entanglement modulus *G_e_* or the zero-rate viscosity *η*_0_. The values that were found to describe the data best are *τ_d_* = 11 s and *η*_0_ = 68 kPa.s. The fitting is particularly good for small frequencies, which is the expected behavior. This is particularly pleasing given the use of only one mode in the model and the neglect of fast (Rouse) mode contributions. The discrepancy between the model and the experimental measurements at larger frequencies can be attributed, as is well known, to the neglect of some other equilibrium relaxation mechanisms, such as CLFs [38,39].

**Figure 6:**
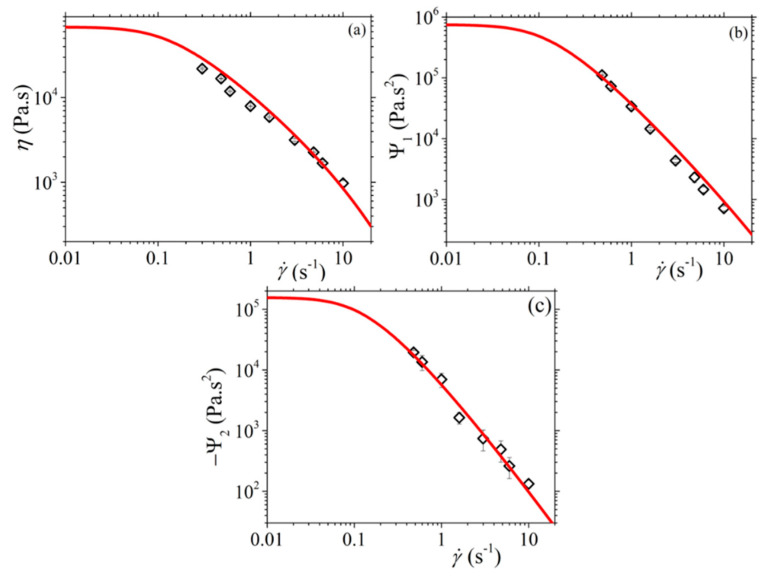
Comparison of the model predictions with the experimental data considered in Ref. [17] for: (**a**) the shear viscosity, (**b**) the first normal stress coefficient, and (**c**) the second normal stress coefficient, at steady state. Model parameters: α = 0.4, *b_e_* = 54, *β_ccr_* = 0.06, and *ξ* = 0.03 (the rest are the same as in Figure 5).

**Figure 7:**
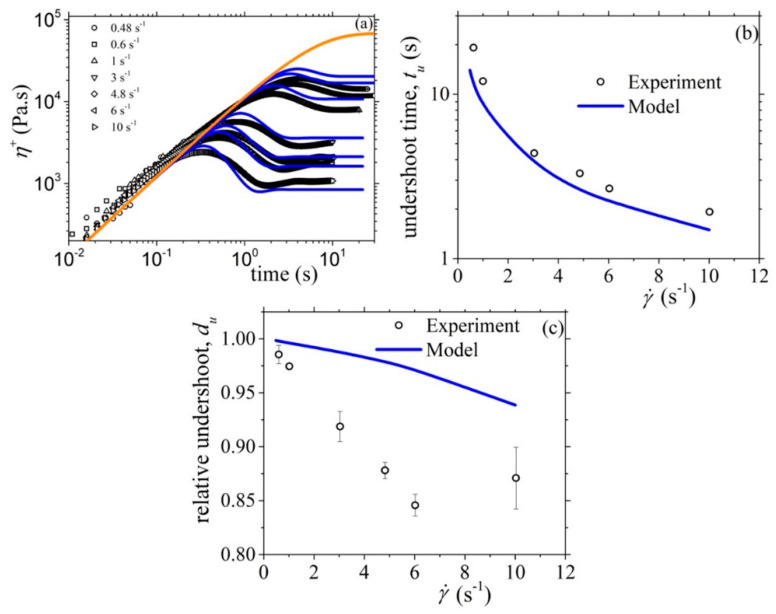
Comparison of the model predictions with the experimental data considered in Ref. [17] for: (**a**) the growth of the shear viscosity, (**b**) the undershoot time, and (**c**) the relative undershoot, upon inception of shear flow. The thick orange line in Figure 7a depicts the LVE envelope, Equation (15a). Same parameter values as in Figure 6.

**Figure 8:**
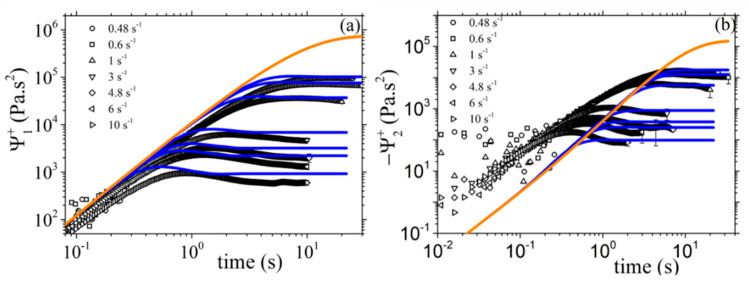
Comparison of the model predictions with the experimental data of Ref. [17] for the growth of the first (**a**) and second (**b**) normal stress coefficients, upon inception of shear flow. The thick orange lines depict the LVE envelope, Equations (15b) and (15c). Same parameter values as in Figure 6.

Next, in Figure 6, we present the fittings for the steady-state values of the shear viscosity and of the two normal stress coefficients, whereas in Figure 7 and Figure 8, we present the corresponding fittings in the case of the start-up of shear flow. There, the Rouse time was calculated through *τ_R_* = *τ_d_*/3Z and, since the entanglement molecular weight of the PS in the solution is equal to 64.6 kDa (see Table S-1 of Ref. [17]), we take *b_e_* = 260. Then, and in order to choose the values of the rest of the model parameter, we considered the following: (a) the parameter α effectively controls the transient second normal stress coefficient, and (b) the undershoot observed at large shear rates in the transient shear viscosity is highly sensitive to the value of the non-affine parameter *ξ* (see Figure 7b). Thus, we came up with the following values: α = 0.4, *β_ccr_* = 0.06, and *ξ* = 0.03. The graphs show that the model offers an exceptionally good description of all three steady-state viscometric functions. In addition, it can predict quite accurately the growth of the shear viscosity and of the first normal stress coefficient to flow start-up; even the undershoot time position, *t_u_*, in the shear viscosity at large shear rates is predicted quite accurately (Figure 7b). The description of the relative undershoot, *d_u_*, (undershoot depth divided by the steady-state shear viscosity value), on the other hand, is less satisfactory (Figure 7c). Finally, the growth of the first normal stress coefficient in the start-up of shear flow is noted to be adequately predicted (Figure 8a), whereas for the growth of the second normal stress coefficient we note that the prediction comes with an offset, even in the LVE envelope (Figure 8b). We hypothesize that this is due to the consideration of only one mode in the present analysis.

#### 3.2.2. Comparison with the Experimental Data of Costanzo et al.

**Figure 9:**
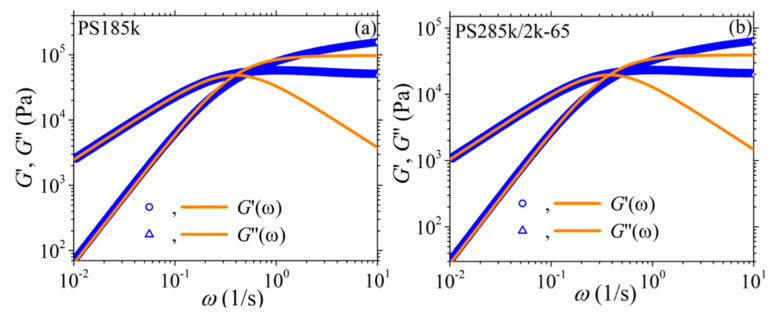
Comparison of the model predictions for the storage and loss moduli with the experimental data of Costanzo et al. [16] for: (**a**) a nearly monodisperse PS melt with *M* = 185 kDa (PS185k) at 160 °C characterized by *τ_d_* = 5.1 s and *η*_0_ = 2.28 × 10^5^ Pa.s, and (**b**) a nearly monodisperse PS solution with *M* = 285 kDa and polymer volume fraction 64.9% (PS285k/2k-65) at 160 °C characterized by *τ_d_* = 5.32 s and *η*_0_ = 1.04 × 10^5^ Pa.s.

**Figure 10:**
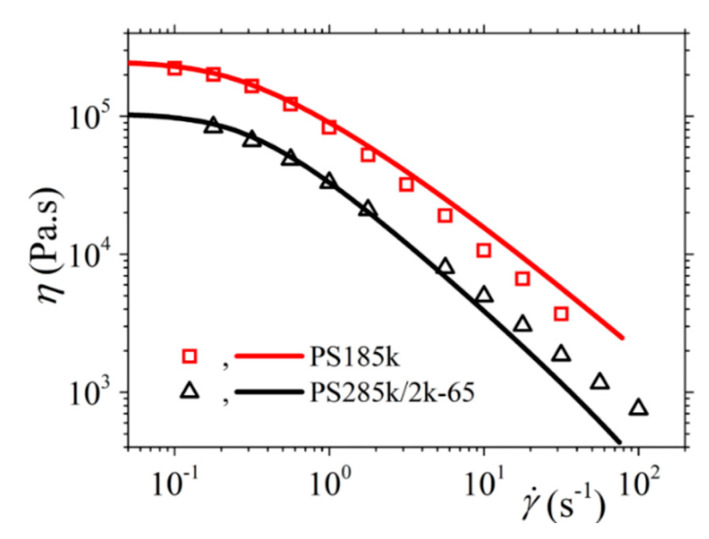
Comparison of the model predictions with the experimental data of Costanzo et al. [16] for the shear viscosity of the PS185k melt and the PS285k/2k-65 solution, at steady state. Model parameters: α = 0.15, *b_e_* = 54, *β_ccr_* = 0.2, and *ξ* = 0 for the melt; α = 0.47, *b_e_* = 83, *β_ccr_* = 0.001, and *ξ* = 0.0015 for the solution (the rest are the same as in Figure 9).

**Figure 11:**
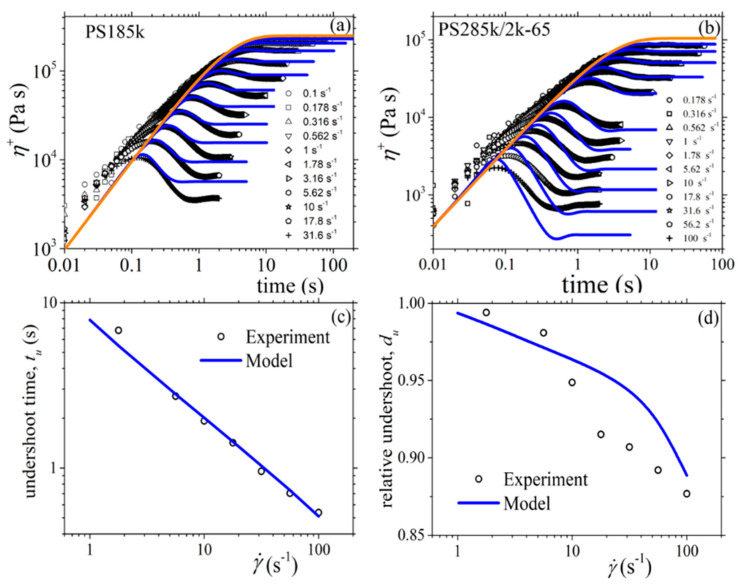
Comparison of the model predictions with the experimental data of Costanzo et al. [16] for: (**a**) the growth of the shear viscosity of the PS185k melt, (**b**) the growth of the shear viscosity of the PS285k/2k-65 solution, (**c**) the undershoot time of the PS285k/2k-65 solution, and (**d**) the relative undershoot of the PS285k/2k-65 solution, upon inception of shear flow. The thick orange line in Figure 11a,b depicts the LVE envelope, Equation (15a). Same parameter values as in Figure 10.

**Figure 12:**
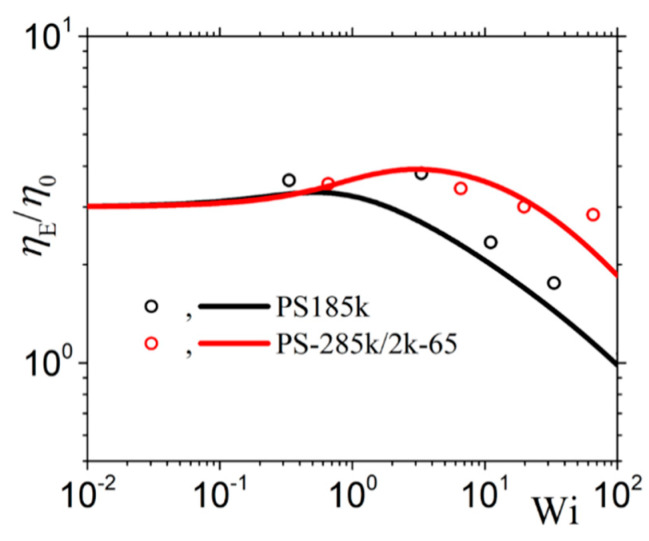
Comparison of the model predictions with the experimental measurements of Costanzo et al. [16] for the steady uniaxial extensional viscosity. Same parameter values as in Figure 11 but in all cases *ξ* = 0.

**Figure 13:**
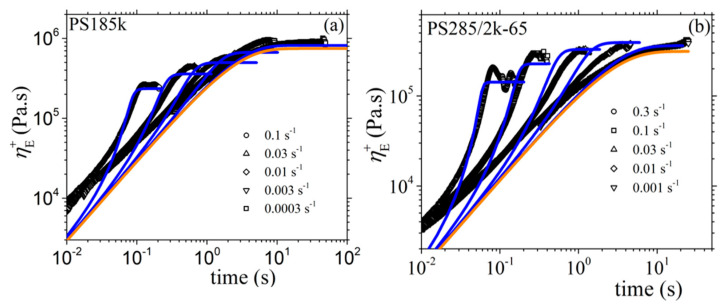
Comparison of the model predictions with the experimental measurements of Costanzo et al. [16] for the extensional stress growth coefficient as a function of time for several stretch rates. The thick orange line depicts the LVE envelope, Equation (17). Same parameter values as in Figure 12.

We next use the new version of our model to describe the experimental data of Costanzo et al. [16] for the polystyrene melt PS185k and the polystyrene solution PS285k/2k-65, both of which have the same number of entanglements, *Z* = 13.9. For the reptation time, we consider the values depicted as *τ_m_* in the Table 2 of Costanzo et al. [16] (5.10 s for the melt, and 5.32 s for the solution). And the same for the Rouse time and the zero-rate viscosity: *τ_R_* = 0.271 s and *η*_0_ = 2.28 × 10^5^ Pa.s for the PS185k melt, and *τ_R_* = 0.319 s and *η*_0_ = 1.04 × 10^5^ Pa.s for the PS285k/2k-65 solution. We then note in Figure 9 the overall agreement between the model predictions and the rheological data for both the melt and the solution despite the use of only one mode; however, we still note deviations at large frequencies as before. Finally, as mentioned before, for the melt *b_e_* = *b_e_* = 54, whereas for the solution *M_e_* = 83 (since 20.5 kDa as calculated through *M_e_*^solution^ = *M_e_*^melt^/*ϕ* with *ϕ* being the polymer volume fraction [16]). Moreover, for the melt, and since no undershoots are observed at large shear rates in the transient shear viscosity, we consider *ξ* = 0. For the remaining parameters, both for the melt and the solution, we follow the same strategy as above, the only difference being that now we use the model to also fit the steady-state and transient elongational viscosities for the two systems. Overall, the following model parameters are used: α = 0.15, *β_ccr_* = 0.2, and *ξ* = 0 for the melt; and α = 0.47, *β_ccr_* = 0.001, and *ξ* = 0.0015 for the solution. Again, the model can describe very well the steady-state (Figure 10) and transient (Figure 11) shear viscosity, and the steady-state (Figure 12) and transient (Figure 13) elongational viscosity for both the PS185k melt, and the PS285k/2k-65 solution. Note also that in the case of the elongational flow, we have used *ξ* = 0 both for the melt and the solution, since no chain tumbling occurs in extensional flows due to their irrotational character. Again, the description of the growth of the shear viscosity is quite gratifying, especially of the position of the undershoot at large shear rates in the case of the polymer solution (Figure 11c); the relative undershoot, on the other hand, is described again less accurately (Figure 11d). Overall, the revised model, even with the use of only a single mode, can quantitatively capture the experimental trends both for entangled polymer melts and concentrated polymer solutions.

## 4. Conclusions

Some time ago, we presented a reformulation of the Marrucci-Ianniruberto constitutive equation [14] for the rheology of entangled polymer melts and concentrated solutions in the context of non-equilibrium thermodynamics [25]. However, rather large values of the CCR parameter had to be invoked for the model not to violate the second law of thermodynamics. Here, we have presented an appropriate modification that avoids the splitting of the evolution equation for the conformation tensor into an orientation and a stretching part; as a result, thermodynamic admissibility allows for a friendlier range of values of the CCR parameter to be used (they must only be non-negative). In addition, and in view of recent experimental evidence for the appearance of undershoots in the transient stress (following the overshoot) at high shear rates whose origin may be traced back to molecular tumbling at high shear rates, the new model contains additional terms accounting for non-affine chain deformation through a slip parameter.

The capability of the new model to describe very satisfactorily rheological data was demonstrated by using it to fit available experimental data for the transient and steady-state shear and elongational rheology of entangled PS melts and solutions. Remarkable success was observed for all viscometric functions checked, considering in particular the use of only a single mode. Given its simplicity and thermodynamic rigor (proven thermodynamic admissibility), this is a remarkable feature of the new model. At the same time, it can also predict quite accurately the elongational rheological data. It can thus be used as an excellent alternative to other successful constitutive models, such as the Rolie-Poly [26] for characterizing the rheological response of polymeric fluids or for carrying out large scale numerical calculations of complex viscoelastic flows.

Of course, the new version of the model is far from being considered complete. There is a few more features and mechanisms of relevance to the flow of entangled polymer fluids that must be accounted for in the corresponding constitutive and transport equations. First, we certainly need to include in the model some additional relaxation mechanisms that were neglected in the present treatment (such as CLFs), in combination with a more rigorous treatment of CR effects. Second, we mention the need to implement a configuration dependent friction coefficient as has been proposed by Mead et al. [40], as well as the proper extension to a multi-mode formulation. Third, and given the wealth of information provided by recent NEMD studies [6,7,8,15] at the level of individual chains and their conformations, it would be highly desirable to check how well the model can describe the conformational properties of entangled polymer melts under flow (and not just the viscometric functions). For example, Sefiddashti et al. [9] have already carried out such a first MD test of several constitutive models. In turn, such a direct comparison would help fully parameterize the model on the outcome of these direct NEMD simulations. It is by bringing together simulations and theory that one can achieve a more complete description of polymer flows, which can then be encoded in the form of friendly and easy-to-use, yet powerful and accurate constitutive laws for the subsequent use in numerical calculations of viscoelastic flows through geometries of practical relevance. Finally, it would be of interest to solve the model in the case of a large amplitude oscillatory shear (LAOS) flow to check how well it can address the nonlinear viscoelastic oscillatory response of entangled polymers [41].

## Appendix A

In this Appendix, we discuss the thermodynamic admissibility of the new model. To this, we need first to guarantee that the conformation tensor is positive-definite. A sufficient (but not necessary) condition for $\tilde{c}$ to be positive definite is that the prefactor of the unit tensor in the evolution equation must be positive [27,42]. This requires that:

(A1) $$\frac{1-\alpha \left(1-\xi \right)}{\tau_{ccr}}>0$$

Since the CCR relaxation time is always positive (see also below), then the new model guarantees a positive definite conformation tensor (non-negative eigenvalues) provided that α < 1/(1 − ξ). Thermodynamic admissibility further requires that the model be compatible with the 2nd law of thermodynamics, according to which the total rate of entropy production within the system must be non-negative. For incompressible and isothermal flows, such a requirement is expressed as [*H_m_*,*H_m_*] ≤ 0 [27], for which a necessary and sufficient condition to hold for the revised model is:

(A2) $$\frac{\delta H_{m}}{\delta {\tilde{c}}_{\alpha \beta }}\Lambda_{\alpha \beta \gamma \varepsilon }\frac{\delta H_{m}}{\delta {\tilde{c}}_{\gamma \varepsilon }}\geq 0$$

This implies that

(A3a) $$\begin{array}{l} \frac{\delta \tilde{A}}{\delta {\tilde{c}}_{\alpha \beta }}\left(\Lambda_{\alpha \beta \gamma \varepsilon }^{\tau_{d}}+\Lambda_{\alpha \beta \gamma \varepsilon }^{\tau_{R}}\right)\frac{\delta \tilde{A}}{\delta {\tilde{c}}_{\gamma \varepsilon }}\geq 0\Rightarrow \\ \frac{1}{2\tau_{ccr}\left(\mathrm{tr}\tilde{c}\right)}\left\{\left[h^{2}\mathrm{tr}\tilde{c}-6h+\mathrm{tr}{\tilde{c}}^{-1}\right]+\alpha \left(1-\xi \right)\left[h^{3}\mathrm{tr}{\tilde{c}}^{2}-3h^{2}\mathrm{tr}\tilde{c}+9h-\mathrm{tr}{\tilde{c}}^{-1}\right]\right\}\geq 0 \end{array}$$

which can also be written as

(A3b) $$\frac{1}{2\tau_{ccr}}\sum_{i=1}^{3}\frac{{\left(h\mu_{i}-1\right)}^{2}}{\mu_{i}}\left\{1+\alpha \left(1-\xi \right)\left(h\mu_{i}-1\right)\right\}\geq 0$$

where *μ_i_*, *i* = {1,2,3} denote the eigenvalues of the conformation tensor. Given that the conformation tensor is positive-definite (thus, *μ_i_* ≥ 0, ∀*i*), the sum in Equation (A3b) is always non-negative. On the other hand, the CCR relaxation time, i.e., the prefactor in Equation (A3b), is non-negative provided that:

(A4) $$\frac{1}{\tau_{ccr}\left(\mathrm{tr}\tilde{c}\right)}\geq 0\Rightarrow \left(\frac{1}{\tau_{R}}-\frac{2}{\tau_{d}}\right)\frac{\beta_{ccr}\left[h\left(\mathrm{tr}(\tilde{c}-I)\right)\mathrm{tr}\tilde{c}-3\right]}{3+\beta_{ccr}\left[h\left(\mathrm{tr}(\tilde{c}-I)\right)\mathrm{tr}\tilde{c}-3\right]}\geq 0$$

Note that both the reptation and Rouse times are positive quantities. First, we need to check whether *τ_d_* > 2*τ_R_*. Since a relation of the form *τ_d_* = 3*Zτ_R_* with *Z* > 1 (otherwise, the polymeric system studied is below the entanglement threshold) is usually employed, this condition is always true. Second, we note that the fraction in Equation (A4) is always positive (it ranges between 0 and unity) for all non-negative values of the CCR parameter. Thus, thermodynamic admissibility is guaranteed, provided that *β_ccr_* ≥ 0.
